# The MTego trap: a potential tool for monitoring malaria and arbovirus vectors

**DOI:** 10.1186/s13071-023-05835-9

**Published:** 2023-06-27

**Authors:** Masudi Suleiman Maasayi, Jane Johnson Machange, Dismas S. Kamande, Ummi Abdul Kibondo, Olukayode G. Odufuwa, Sarah Jane Moore, Mgeni Mohamed Tambwe

**Affiliations:** 1 Vector Control Product Testing Unit, Environmental Health and Ecological Science Department, Ifakara Health Institute, P.O. Box 74, Bagamoyo, Tanzania; 2 School of Life Sciences and Bioengineering, The Nelson Mandela African Institution of Science and Technology (NM-AIST), P.O. Box 447, Arusha, Tanzania; 3 London School of Hygiene and Tropical Medicine, Keppel Street, London, WC1E 7HT UK; 4 Vector Biology Unit, Epidemiology and Public Health Department, Swiss Tropical and Public Health Institute, Kreuzstrasse 2, Allschwil, 4123 Basel, Switzerland; 5 University of Basel, Petersplatz 1, 4001 Basel, Switzerland

**Keywords:** MTego, BG-Pro trap, Human landing catch, Trap, Odour-baited trap, Mosquito, *Anopheles*, *Culex*, *Aedes*

## Abstract

**Background:**

Odour-baited traps are useful for vector surveillance and control. However, most existing traps have shown inconsistent recapture rates across different mosquito species, necessitating the need for more effective and efficient traps. The MTego trap with integrated thermal stimuli has been developed as an alternative trap. This study was undertaken to determine and compare the efficacy of the MTego trap to that of the Biogents (BG) modular BG-Pro (BGP) trap for sampling different mosquito species in a semi-field system.

**Methods:**

Fully balanced Latin square design experiments (no-choice and dual choice) were conducted in semi-field chambers using laboratory-reared female *Anopheles gambiae* sensu stricto, *Anopheles funestus*, *Anopheles arabiensis*, *Culex quinquefasciatus* and *Aedes aegypti*. There were 16 replicates, and 50 mosquitoes of each species were released in each chamber per replicate. The evaluated traps were as follows: the MTego trap baited with PM6 (MT-PM6), the MTego trap baited with BG-Lure (BGL) (MT-BGL), and the BGP trap baited with BG-Lure (BGP-BGL).

**Results:**

In the no-choice test, the MT-BGL and BGP-BGL traps captured a similar proportion of *An. gambiae* (31% vs 29%, *P*-value = 0.519) and *An. funestus* (32% vs 33%, *P* = 0.520). The MT-PM6 and BGP-BGL traps showed no significant difference in capturing *Ae. aegypti* (33% vs 31%, *P* = 0.324). However, the BGP-BGL caught more *An. arabiensis* and *Cx. quinquefasciatus* mosquitoes than the other traps (*P* < 0.0001). In the dual-choice test of MT-PM6 vs BGP-BGL, similar proportions of *An. funestus* (25% vs 27%, *P* = 0.473) and *Ae. aegypti* (29% vs 25%, *P* = 0.264) were captured in the traps, while the BGP-BGL captured more *An. gambiae*, *An. arabiensis* and *Cx. quinquefasciatus* mosquitoes than the MT-PM6 (*P* < 0.0001).

**Conclusions:**

This study demonstrated that the MTego trap has potential as a tool that can be used interchangeably with the BGP trap for sampling anthropophilic mosquitoes including African malaria vectors *An. gambiae* and *An. funestus* and the principal arbovirus vector *Ae. aegypti*.

**Graphical abstract:**

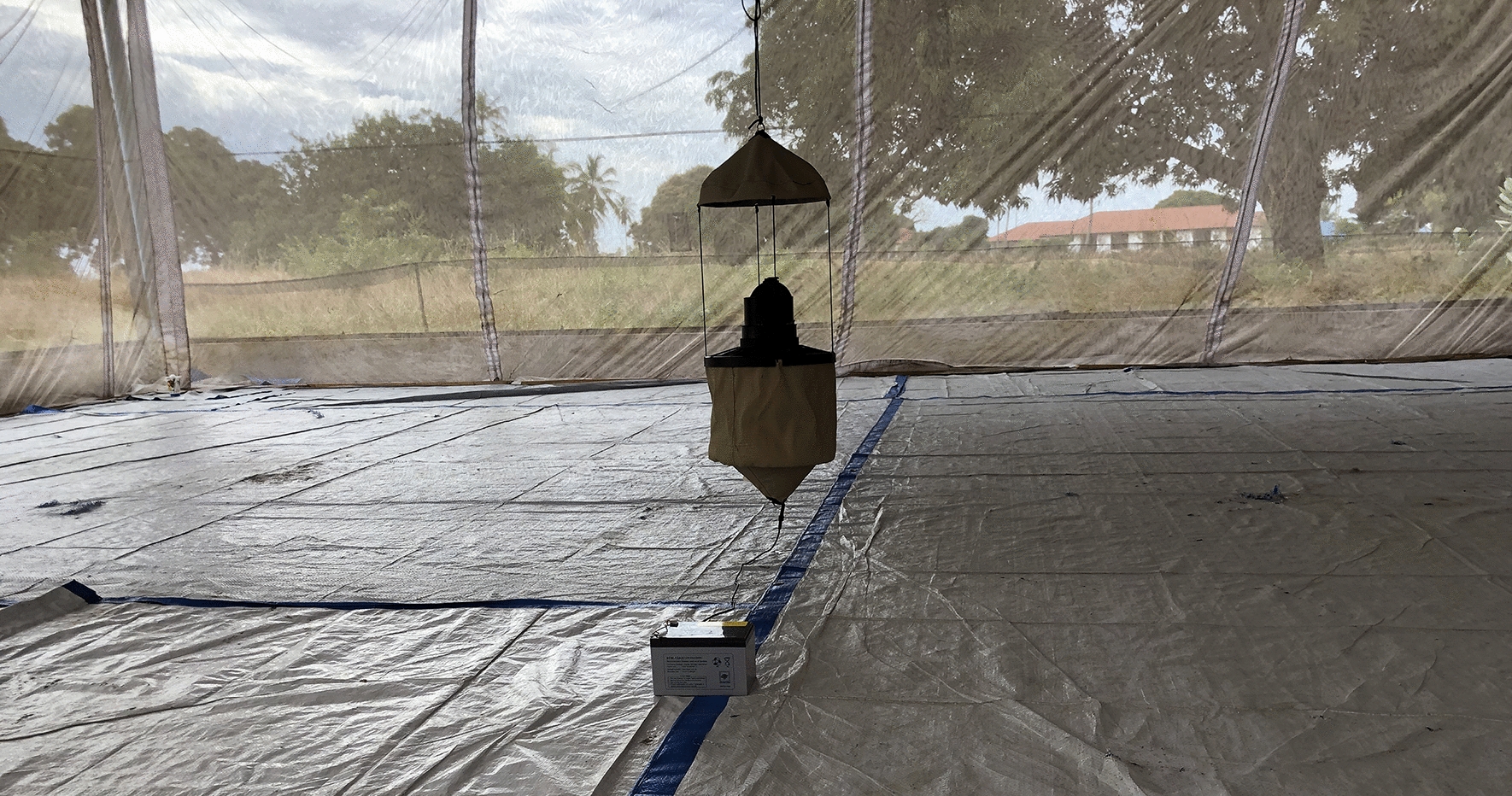

## Background

Mosquitoes are vectors of many diseases, including malaria, dengue fever, Zika and West Nile virus [1]. Transmission of these diseases occurs when infected mosquitoes successfully locate, land on, and blood feed on vertebrate hosts to obtain proteins needed to nourish their eggs [2]. Host-seeking behaviour in mosquitoes is governed by a combination of visual and physical cues together with chemical cues, which are detected using olfactory receptors located on the antennae [3, 4]. Carbon dioxide (CO_2_) acts as a long-range signal that alerts mosquitoes to the presence of a vertebrate host and sensitises them to respond to other host olfactory cues [3]. After a mosquito has oriented towards the host, other cues including skin odours, heat and moisture are used to stimulate landing and feeding [5, 6].

Odour-baited traps (OBTs) take advantage of this behaviour by using specific odours, in addition to manipulative physical and visual cues, to attract mosquitoes [7–10]. Thus, traps are suggested as potential options for integrated vector management due to their proven effect in reducing populations of adult mosquitoes and controlling diseases in various settings [11–14]. For example, a cluster randomised controlled trial in Brazil demonstrated that mass trapping with Biogents (BG) BG-Sentinel traps reduced the population of *Aedes aegypti* and dengue incidence [15]. Additionally, a stepped wedge cluster randomised trial in Kenya reported a substantial reduction of the *Anopheles funestus* population and malaria prevalence in areas where homes were installed with Suna traps compared to the non-intervention areas [16]. Furthermore, Jahir et al. [17] recently demonstrated that BG-Mosquitaire traps distributed at higher densities when used in combination with larval source management drastically reduced populations of *Aedes* and *Culex* mosquitoes by 93–98% in small Maldivian islands.

While OBTs have been shown to be promising in reducing mosquito populations, the performance of most existing traps has been inconsistent for different mosquito species and geographical locations, necessitating the need for more effective and efficient traps. The MTego trap has been developed as an alternative OBT. In addition to the chemical and visual cues that are normally used in OBTs, heat and moisture are included as additional stimuli in the MTego to improve trap capture. A previous study [18] showed that the MTego trap was highly effective at sampling *Anopheles gambiae* mosquitoes, outperforming the BG-Suna trap in both laboratory and semi-field environments. However, its efficiency for other mosquito species remains unknown. Therefore, this study was undertaken to determine and compare the efficacy of the MTego trap relative to the BGP trap for sampling adult mosquitoes of the genera *Anopheles*, *Culex* and *Aedes* in a semi-field system (SFS) in Tanzania. In addition, the performance of these traps was compared to the human landing catch (HLC) method, which is considered the standard method for sampling human-biting mosquitoes [19].

## Methods

### Study area

The study was conducted in a large SFS located at Ifakara Health Institute (IHI) in Bagamoyo district, Tanzania. The SFS measures 29 × 21 × 4.5 m, is screened with shade mesh walls, and has a polyethene roof mounted on an elevated concrete platform. It is divided into two compartments, each measuring 29 × 9 m, with a middle buffer chamber. Using polyethene sheathing and netting cages, the compartments can be further divided into smaller independent chambers to suit the needs of a particular study.

### Mosquitoes

Laboratory-reared *Anopheles gambiae* sensu stricto (s.s.) (Ifakara strain), *Anopheles funestus* (Fumoz strain)*, Anopheles arabiensis* (Kingani strain), *Culex quinquefasciatus* (Bagamoyo strain) and *Ae. aegypti* (Bagamoyo strain) mosquitoes aged 3–5 days were used in the experiments. Mosquitoes are reared at the insectary at 27 °C ± 5 °C and 70 ± 20% relative humidity (RH) and ambient 12:12 light:dark, following MR4 guidelines [20]. Larvae are nourished with Tetramin fish food, while adult mosquitoes have unrestricted access to a 10% sucrose solution for sustenance. To stimulate egg production in adult females, cow blood meals (heparinised) are provided through a membrane-feeding assay. The mosquitoes were blood naive and sugar starved for 6–10 h before the experiments. *Anopheles arabiensis* mosquitoes were marked with fluorescent dye to distinguish their strains from *An. gambiae* s.s. Previous experiments showed that colour pigments do not significantly affect mosquito survival or host preference [21].

### Tested traps and HLC method

#### MTego trap

**Fig. 1 a–c:**
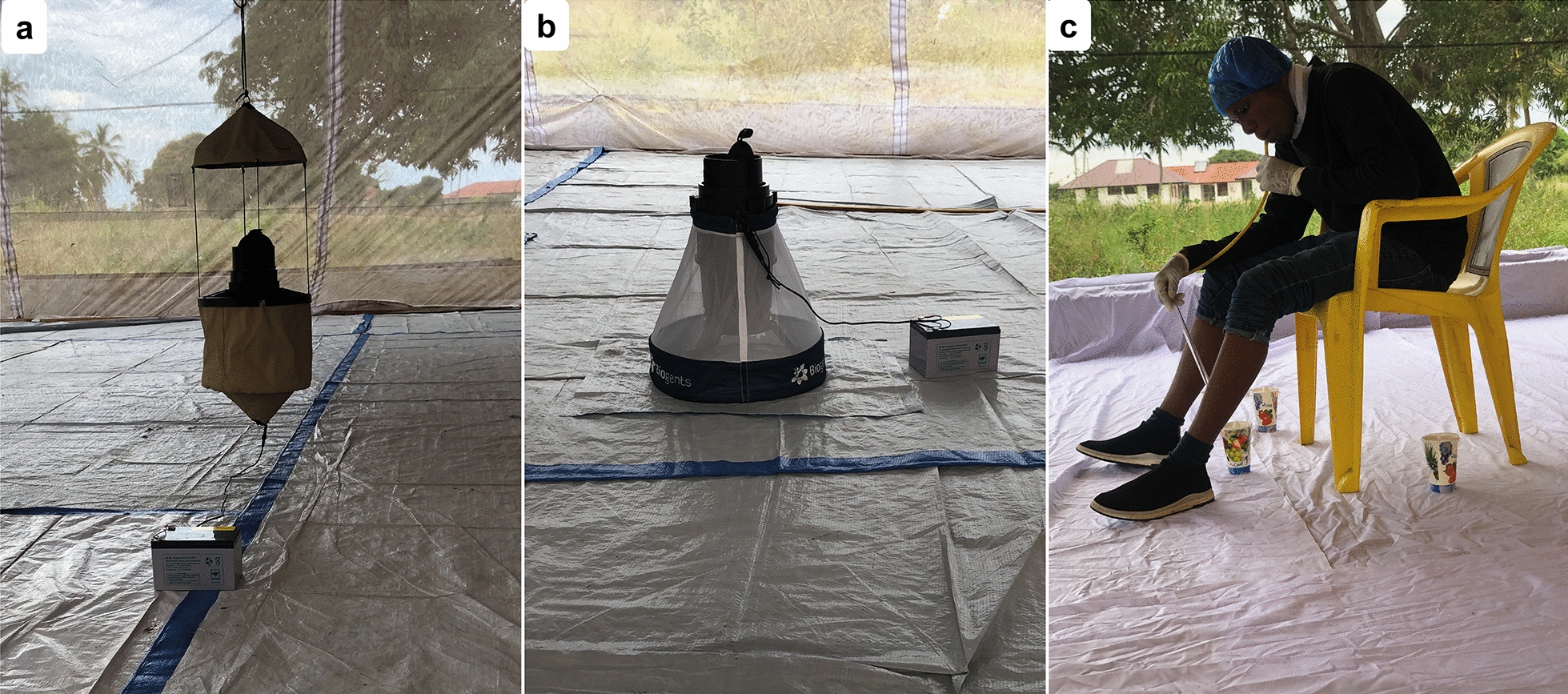
Mosquito sampling methods evaluated in the study. **a** The MTego trap. **b** The Biogents (BG) BG-Pro (BGP) trap. **c** A volunteer conducting human landing catches (HLCs)

The MTego (PreMal BV, The Netherlands) is a novel mosquito trap that uses a counterflow principle and a brushless 12-V direct current fan to capture mosquitoes. The trap uses baits that attract mosquitoes, generates heat to mimic that of a human body through a wrapped low-powered heating element at the base of its inlet, and generates moisture using warm water that is added to the ripstop nylon bag before operation [18]. The trap has a foldable ripstop nylon bag and an insect net on top that allows the circulation of odour-saturated air. The trap also has an inlet module with an integrated catching cage for easy removal of caught mosquitoes. The trap was assembled according to the manufacturer’s instructions and hung 10 cm from the ground (Fig. 1a); 250 ml of warm water was poured into the bag at the start of each experiment.

#### BG-Pro trap

The BG-Pro (BGP; Biogents, Regensburg, Germany) is a cone-shaped fabric trap that uses a three-blade fan to generate airflow which sucks in mosquitoes that are close to the inlet funnel. The trap also uses bait, such as BG-Lure (BGL; Biogents), to attract mosquitoes. It can be powered by a 5-V alternating current power bank or a 6-V direct current battery. The trap is collapsible and comes equipped with a ultraviolet-light-emitting diode light, rain cover and internal tripod, and can be configured to hang from a hook on the ceiling or to stand on the ground. The trap is smaller and more portable than similar traps that use traditional batteries [22]. The trap was assembled according to the manufacturer’s instructions, powered by a 12-V battery and stood directly on the ground (Fig. 1b).

#### Human landing catch

HLC is a standard mosquito sampling method that requires an adult volunteer to sit on a chair and collect any mosquitoes that land on their exposed legs by aspirating them with a mouth aspirator [19]. One adult male volunteer, fully trained and voluntarily recruited through written informed consent, conducted the HLC. Mosquitoes were aspirated as they landed on his exposed legs (Fig. 1c). The captured mosquitoes were kept in paper cups that were exchanged for fresh cups after each hour, and transferred to the insectary after the experiment.

### Odour blends

#### BG-Lure

The BG-Lure is a blend of chemicals composed of ammonia solution, (*S*)-lactic acid and caproic acid. The lure is designed to mimic the scent of human skin and other compounds that are attractive to mosquitoes [10].

#### PreMal 6 lure

The PreMal 6 lure (PM6) (PreMal BV, The Netherlands) is a synthetic attractant which is used to imitate evaporated human sweat, leading to better capture rates in the MTego trap. The scent is dispensed using a sachet that is suspended inside the trap. The sachet slowly releases the scent to produce sustained effectiveness for up to 90 days before requiring replacement [23].

### Study procedures

#### Experiment 1. Trapping efficacy of the MTego traps, BGP trap and HLC in the no-choice test

**Fig. 2 a, b:**
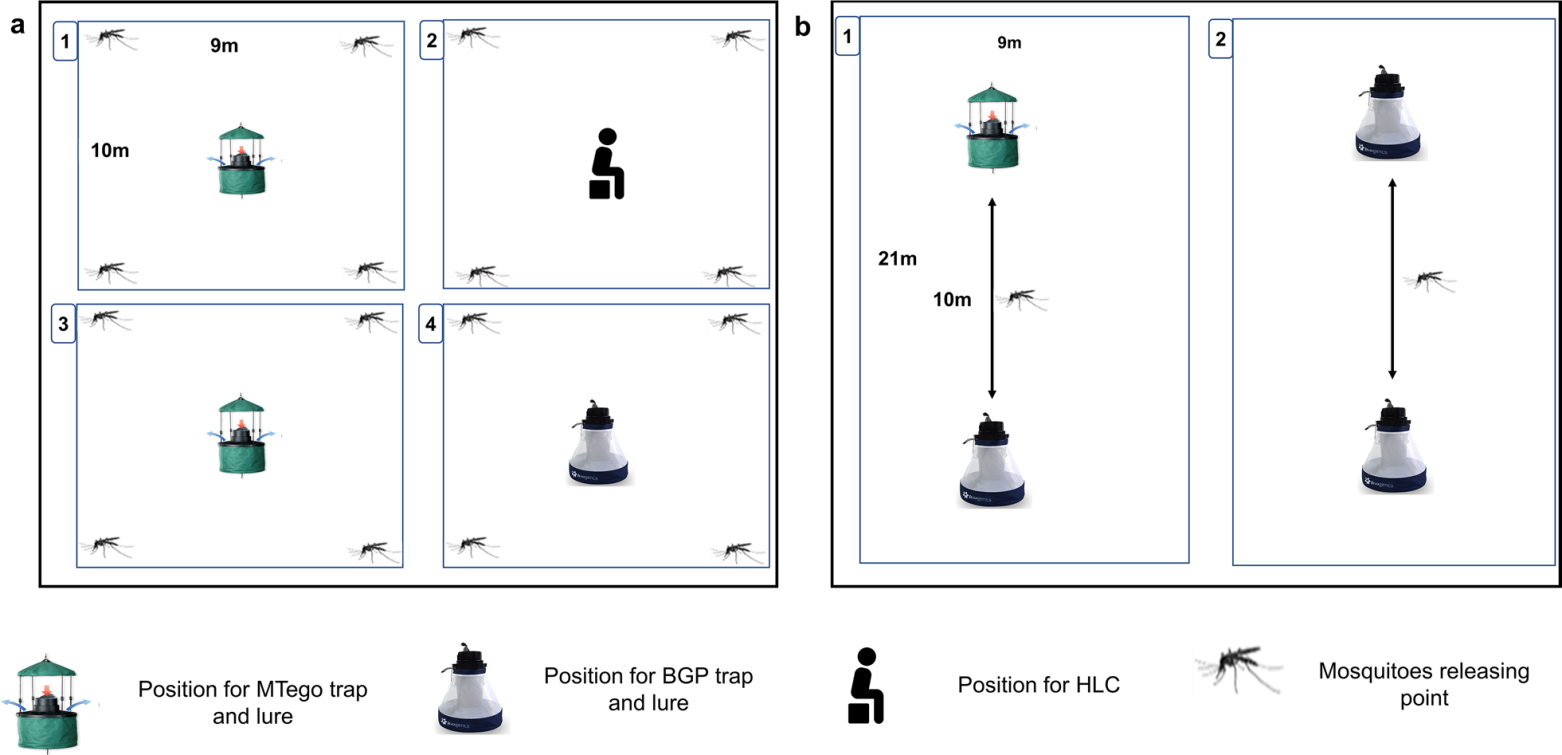
Schematic representation of the experiments in the semi-field system (SFS). **a** The no-choice test to compare the MTego traps, BGP trap and HLC. **b** The dual-choice test to compare the MTego trap and BGP trap. The numbers in the boxes indicate the chambers in the SFS. For other abbreviations, see Fig. 1

A 4 × 4 Latin square design experiment was conducted to evaluate the trapping efficacy of the MTego trap baited with PM6 (MT-PM6), the MTego trap baited with BG-Lure (MT-BGL), the BGP trap baited with BG-Lure (BGP-BGL) and HLC. The SFS was divided into four chambers with polyethene fabric and a large netting cage measuring 10 × 9 m was installed in each. The trapping methods were assigned randomly to each chamber on the first day of the experiment and sequentially rotated daily in a Latin square pattern across the chambers such that after 16 days of experimentation each method had been tested in each chamber four times. Test mosquitoes were acclimatised in the middle compartment for 45 min before the experiment began. A total of 50 mosquitoes of each species were simultaneously released [24] into each chamber from four releasing points (Fig. 2a). For *Ae. aegypti*, the experiment commenced at 1600 hours to accommodate its biting behaviour, which primarily occurs during daylight hours. For *Anopheles* and *Culex* mosquitoes, the experiment started at 1830 hours and continued throughout the night to cover their active host-seeking period. The traps operated from 1600 to 0700 hours the next morning, while HLCs were conducted from 1600 to 2200 hours with a total break period of 60 min (a 30-min break between 1800 and 1830 hours and a 10-min break after each successive hour). Trapped mosquitoes were refrigerated, identified, and manually counted. After every experiment, the SFS was thoroughly cleaned and searched for remaining mosquitoes using a Prokopack aspirator (John W Hock, Gainesville, FL). The traps were also cleaned using 70% ethanol and dried outdoors before they were reused.

#### Experiment 2. Trapping efficacy of the MTego trap relative to the BGP trap in the dual-choice test

A 2 × 2 balanced Latin square design experiment was conducted to compare the trapping efficacy of the MT-PM6 relative to the BGP-BGL. The SFS was divided into two chambers with polyethene fabric, and a large netting cage measuring 20 × 9 m was installed in each. The MT-PM6 was placed 10 m from the BGP-BGL in one chamber, and two BGP-BGL were positioned 10 m apart from one another in the other chamber (Fig. 2b). Fifty mosquitoes of each species were released at the centre of each chamber. The experiment was conducted for 16 replicates in which the traps were rotated daily across the positions in a sequential Latin square design. Other experimental procedures were maintained as in the previous experiment.

### Data analysis

Data were double-entered in Microsoft Excel 2021 and analysed using STATA 17 [25]. Descriptive statistics were conducted to estimate the mean percentage and 95% confidence intervals (CI) of each mosquito species captured in each trap. In the no-choice experiment, multilevel mixed-effects logistic regression following binomial distribution and logit function was used, while a multilevel mixed-effects generalised linear model with a negative binomial error and log link function was used to model the count data in the dual-choice experiment. In both analyses, the fixed effects were trap, position and chamber, while day was included as a random effect.

## Results

### Experiment 1. Trapping efficacy of the MTego traps, BGP trap and HLC in the no-choice test

**Fig. 3:**
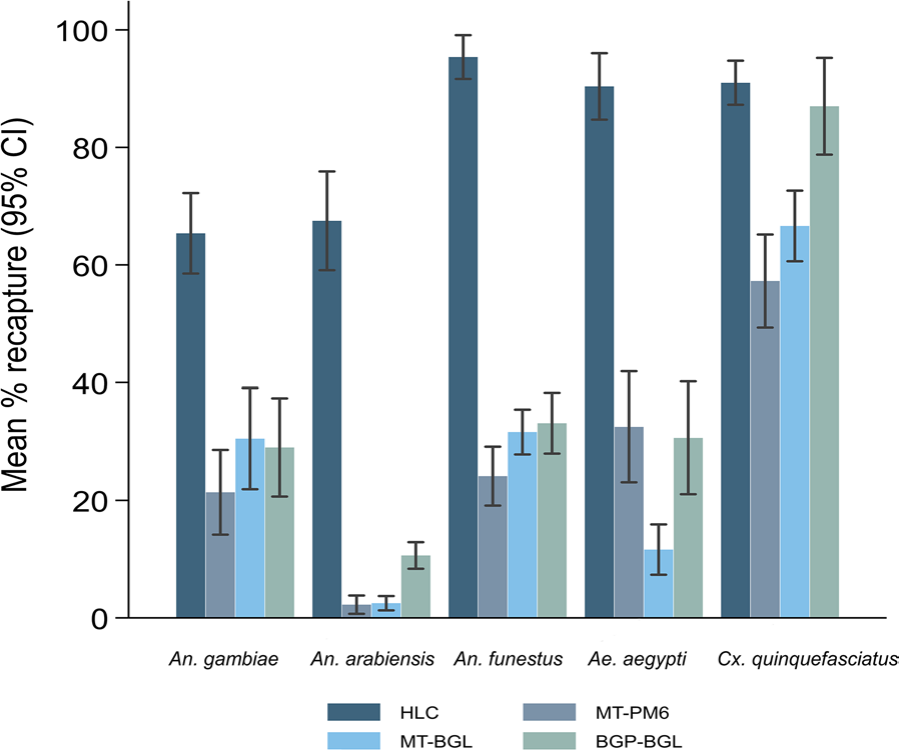
Percentages of mosquitoes recaptured by the MTego traps, BGP trap and HLC in the no-choice test.* CI* Confidence interval, *MT-BGL* MTego trap baited with BG-Lure, *MT-PM6* MTego trap baited with PM6; for other abbreviations, see Fig. 1

The average environmental conditions throughout the experiment were 23 °C (21–26 °C) and 82% (70–92%) RH. In total, approximately 3200 mosquitoes of each species were released in the SFS, of which 1170 (37%) *An. gambiae*, 1474 (46%) *An. funestus*, 663 (21%) *An. arabiensis*, 1321 (41%) *Ae. aegypti* and 2415 (75%) *Cx. quinquefasciatus* were recaptured by the traps. Overall, HLC was the most efficient method for collecting all of the mosquito species, while the traps varied in their performance depending on the species. *Anopheles arabiensis* showed a lower response to all of the traps, whereas *Cx. quinquefasciatus* showed a higher response to all of the traps, especially BGP-BGL, which nearly matched that of the HLC (Fig. 3; Table 1).

**Table 1:** Relative trapping efficacy of the MTego traps, Biogents (BG) Pro (BGP) trap and human landing catch (*HLC*) method in the no-choice test

Mosquito species	Trapping method	Total catch	Mean % (CI)	OR (95% CI)
*Anopheles gambiae*	BGP-BGL	232	29.0 (20.7–37.4)	Ref.
	MT-PM6	171	21.4 (14.2–28.9)	0.66 (0.52–0.83)
	MT-BGL	244	30.5 (21.9–39.1)	1.07 (0.86–1.34)†
	HLC	523	65.4 (58.5–72.2)	4.88 (3.94–6.07)
*Anopheles arabiensis*	BGP-BGL	85	10.6 (8.4–12.9)	Ref.
	MT-PM6	18	2.3 (0.7–3.8)	0.19 (0.11–0.32)
	MT-BGL	20	2.5 (1.3–3.7)	0.21 (0.13–0.35)
	HLC	540	67.0 (59.1–75.9)	19.74 (14.90–26.14)
*Anopheles funestus*	BGP-BGL	265	33.1 (27.7–38.3)	Ref.
	MT-PM6	193	24.1 (19.1–29.1)	0.64 (0.52–0.80)
	MT-BGL	253	31.6 (27.8–35.4)	0.93 (0.76–1.15)†
	HLC	763	95.4 (91.7–99.1)	41.84 (29.14–60.08)
*Aedes aegypti*	BGP-BGL	245	30.6 (21.0–40.2)	Ref.
	MT-PM6	260	32.5 (23.1–41.9)	1.12 (0.90–1.40)†
	MT-BGL	93	11.6 (7.3–15.91)	0.28 (0.21–0.36)
	HLC	723	90.4 (84.7–96.0)	33.47 (24.29–46.11)
*Culex quinquefasciatus*	BGP-BGL	696	87.0 (78.8–95.2)	Ref.
	MT-PM6	458	57.3 (49.3–65.2)	0.18 (0.14–0.24)
	MT-BGL	533	66.6 (60.6–72.6)	0.28 (0.22–0.36)
	HLC	728	91.0 (87.2–94.8)	1.52 (1.10–2.10)*

The odds ratios (*OR*) were derived from multilevel mixed-effects logistic regression with a binomial distribution and logit function. Trap type, chamber and position were adjusted for fixed effects, and day was a random effect

*CI* Confidence interval, *BGP-BGL* BGP trap baited with BG-Lure, *MT-PM6* MTego trap baited with PM6, *MT-BGL* MTego trap baited with BG-Lure, *HLC* human landing catch,* Ref.* reference

†* P* > 0.32, * *P* = 0.011; all other tests, *P* < 0.0001

Mosquito responses to the MT-BGL and BGP-BGL traps were similar for *An. gambiae* [OR = 1.07 (95% CI, 0.86–1.34), *P* = 0.519] and *An. funestus* [OR = 0.93 (95% CI, 0.76–1.15), *P* = 0.520]. For *An. arabiensis, Ae. aegypti* and *Cx. quinquefasciatus*, lower responses to MT-BGL relative to BGP-BGL were observed (*P* < 0.0001 for all species) (Table 1).

*Aedes aegypti* showed similar responses to MT-PM6 and BGP-BGL traps [OR = 1.12 (95% CI, 0.90–1.40), *P* = 0.324], while *An. gambiae, An. arabiensis, An. funestus* and *Cx. quinquefasciatus* showed a lower response (Table 1).

### Experiment 2. Trapping efficacy of the MTego trap relative to the BGP trap in the dual-choice test

**Fig. 4:**
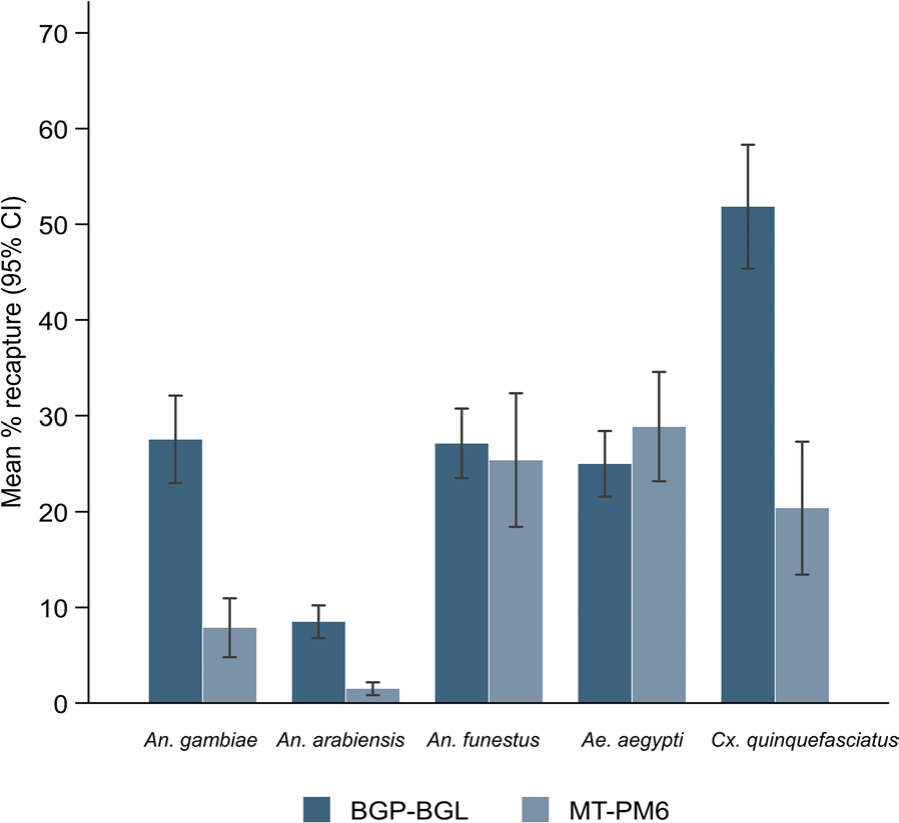
Percentage of mosquitoes recaptured by MTego and BGP traps in the dual-choice test

During the experiment, environmental conditions were 23 °C (22–26 °C) and 78% (61–84%) RH. In total, approximately 1600 mosquitoes of each species were released throughout the experiment, of which 724 (45%) *An. gambiae*, 854 (53%) *An. funestus*, 216 (13.5%) *An. arabiensis*, 831 (51.9%) *Ae. aegypti* and 1407 (87.9%) *Cx. quinquefasciatus* were recaptured by the traps. Overall, the capture rates of traps (combined proportion) were again lower for *An. arabiensis* and several times higher for *Cx. quinquefasciatus* (Fig. 4; Table 2).

**Table 2:** Relative trapping efficacy of the MTego trap compared to the BGP trap in the dual-choice test

Mosquito species	Trapping method	Total catch	Mean % (CI)	IRR (95% CI)
*Anopheles gambiae*	BGP-BGL	661	27.54 (22.99–32.09)	Ref.
	MT-PM6	63	7.88 (4.80–10.95)	0.28 (0.19–0.41)
*Anopheles arabiensis*	BGP-BGL	204	8.50 (6.78–10.22)	Ref.
	MT-PM6	12	1.50 (0.83–2.17)	0.18 (0.09–0.33)
*Anopheles funestus*	BGP-BGL	651	27.13 (23.52–30.73)	Ref.
	MT-PM6	203	25.38 (18.41–32.34)	0.93 (0.76–1.14)†
*Aedes aegypti*	BGP-BGL	600	25.00 (21.57–28.43)	Ref
	MT-PM6	231	28.88 (23.18–34.57)	1.14 (0.90–1.45)†
*Culex quinquefasciatus*	BGP-BGL	1244	51.83 (45.35–58.31)	Ref.
	MT-PM6	163	20.38 (13.43–27.32)	0.38 (0.31–0.47)

The incidence rate ratio (*IRR*) was derived from the multilevel mixed-effects generalised linear model with a negative binomial distribution and log link function. Trap type, chamber and position were adjusted for fixed effects, and day was a random effect

† *P * > 0.26; all other tests, *P* < 0.0001

The MT-PM6 and BGP-BGL had similar capture rates for *Ae. aegypti* [incidence rate ratio (IRR) = 1.14 (95% CI, 0.90–1.45), *P* = 0.264] and *An. funestus* [IRR = 0.93 (95% CI, 0.76–1.14), *P* = 0.473] (Table 2). Conversely, MT-PM6 captured significantly fewer *An. gambiae* [IRR = 0.28 (95% CI, 0.19–0.41), *P* < 0.0001], *An. arabiensis* [IRR = 0.18 (95% CI, 0.09–0.33), *P* < 0.0001] and *Cx. quinquefasciatus* [IRR = 0.38 (95% CI, 0.31–0.47), *P* < 0.0001) than the BGP-BGL (Table 2).

## Discussion

The geographic distributions of mosquito-borne diseases are expanding as a result of rapid, unplanned urbanisation and climate change [26]. New and improved methods are needed to control vector populations and the diseases that they transmit. The use of traps in vector surveillance is a popular method for keeping track of the spread, number, and infection levels of vector populations. In this study, the MTego trap with integrated thermal stimuli and the modular BG-Pro trap were explored as alternative devices for monitoring *Anopheles*, *Culex* and *Aedes* mosquitoes.

In a previous study [18], the MTego trap demonstrated 4.7 times greater efficacy at capturing *An. gambiae* than the BG-Suna trap. However, the performance of the MTego trap for different mosquito species depends on the attractant utilised in the trap, as demonstrated in the current study. When baited with BGL, the MTego showed comparable performance to the BGP-BGL at capturing *An. gambiae* and *An. funestus*. In contrast, when augmented with PM6, the trap exhibited high performance at capturing *Ae. aegypti*. These results are consistent with those of previous studies that indicated that bait type [27, 28] and composition and concentration of chemicals in odour blends [29–32] can significantly impact the performance of OBTs. Contrary to the results of the no-choice experiment, the MT-PM6 exhibited a similar level of efficacy to the BGP-BGL at capturing *An. funestus* when the two traps were used simultaneously in the same chamber. Heat and moisture, which were generated by the MTego trap, may have influenced this outcome, as reported in previous studies where mosquitoes tended to show a preference for a human volunteer over traps at short distances apart [8, 33]. Overall, the MTego trap shows potential as a valuable tool for sampling various mosquito species, and its performance can be enhanced by utilising diverse attractants, depending on the targeted species and the prevailing context.

The BGP-BGL displayed comparable efficacy to the MTego traps in capturing the most anthropophilic species tested: *An. gambiae*, *An. funestus* and *Ae. aegypti* (Figs. 3, 4). This is in contrast to the results of Degener et al. [22], who reported that BGP was ineffective for sampling *Anopheles* mosquitoes in a field study in Mozambique. It is likely that local population densities and competing sources of host kairomones may have affected the trap’s performance in the field study.

Overall, the results showed that the capture performances of all of the traps were much lower for *An. arabiensis* than for *An. gambiae*, *An. funestus*, and *Ae. aegypti*, while the response of *Cx. quinquefasciatus* was several times higher than those of all the other mosquito species (Figs. 3, 4). These findings are consistent with those of Mburu et al. [34], who also observed that *An. arabiensis* was less attracted than *An. gambiae* to an MB5-baited MM-X trap, and Kim et al. [32], who reported that *Cx. quinquefasciatus* was more attracted than *Ae. aegypti* to CO_2_-baited BG-Sentinel traps.

It is important to develop species-specific attractants, given that most existing attractants have been optimised for the anthropophilic *An. gambiae* and *Ae. aegypti* and vectors with a wider host preference also transmit diseases. For example, *An. arabiensis* is an opportunistic vector that utilises CO_2_ as a generic host cue and feeds on both humans and animals, while *An. gambiae*, *An. funestus*, and *Ae. aegypti* prefer humans and use CO_2_ together with odorants that are specific to humans for locating hosts [35–37]. *Culex quinquefasciatus* is known to have a high degree of plasticity in its host preferences, which vary from 100% animal feeding to high degrees of preference for birds [36]. The results of the present study add to those of the existing body of work that show that the specific blend of chemicals used in a synthetic bait may be more attractive to certain species of mosquitoes than others.

We also found that higher proportions of all of the mosquito species were collected using HLC than with the MTego or BGP traps. This may have been a consequence of the complexity of human host cues and their dynamic nature in various environments [2, 13]. Although Okumu et al. [8] developed a highly attractive blend, it did not match the attractiveness of humans when the two stimuli were evaluated simultaneously in the same hut. We did not directly compare the attractiveness of the traps to that of humans in the present study, but based on the no-choice results, it is clear that humans remain more attractive to host-seeking mosquitoes than current lures. Similarly, recent studies showed that odour-baited Suna [38] and BG-Sentinel traps [33, 39] were less effective at capturing mosquitoes in the presence of humans. Further research exploring highly effective attractants to enhance trap performance is necessary.

Although the MTego trap incorporates an element to generate heat, and produces moisture through the addition of warm water, it was evident that these supplementary cues did not significantly enhance its performance relative to that of the BGP trap. However, a previous study indicated that the inclusion of heat generation substantially enhanced the efficacy of the MTego trap compared to the Suna trap for *An. gambiae* [18]. Furthermore, we did not examine the other configurations of the BGP trap, which can be changed to give three different types of trap, as described by Degener et al. [22]. These need further investigation, to quantify the effect of their features on overall attraction and catch.

Laboratory-reared mosquitoes offer advantages in terms of standardised conditions and the availability of an adequate number of mosquitoes for experiments. However, it is crucial to consider potential behavioural changes that may arise due to colonisation or long-term laboratory rearing. These changes may distance laboratory-reared mosquitoes from their wild siblings in their behaviour. Consequently, the generalisability of results from semi-field studies to all populations of a target mosquito species may be limited. Different mosquito populations can exhibit distinct behaviours and preferences, which can impact trap performance. To address these concerns, future experiments should include comparative behavioural analysis between laboratory-reared and wild-caught mosquitoes and similar experiments in the field with wild mosquitoes. The results of these types of investigations would bridge the gap between semi-field and field data, enhancing our understanding of mosquito behaviour and the effectiveness of trapping methods in real-world conditions.

Despite the limitations discussed here, the current study demonstrated that OBTs remain useful options for integrated vector management, as they can consistently remove mosquitoes from a population on a daily or nightly basis to the extent that they can have an impact on disease. Before deploying traps in a given setting, it is worth noting that their performance can vary depending on their design, the type of bait used, the setting and the mosquito species being targeted. Therefore, selecting the optimal trap-lure combination for a specific setting should maximise trap efficiency.

## Conclusions

This study demonstrated that the MTego trap has potential as a tool that can be used interchangeably with the BGP trap for sampling anthropophilic mosquitoes including African malaria vectors *An. gambiae* and *An. funestus* and the principal arbovirus vector *Ae. aegypti*. The traps tested here caught a substantial proportion of the released mosquitoes in a simulated outdoor setting and may be used outdoors for sampling a variety of mosquito species, including those of the genera *Anopheles*, *Culex* and *Aedes*.

## Data Availability

The data generated during this study are available from the corresponding author on reasonable request.

## Abbreviations

BG: Biogents
BGL: BG-Lure
BGP: BG-Pro trap
BGP-BGL: BG-Pro trap baited with BG-Lure
HLC: Human landing catch
MT-BGL: MTego trap baited with BG-Lure
MT-PM6: MTego trap baited with PreMal 6 lure
OBTs: Odour-baited traps
PM6: PreMal 6 lure
SFS: Semi-field system
